# Crystal structure of di­chlorido­bis­(1,3-diisopropyl-4,5-dimethyl-2*H*-imidazole-2-thione-κ*S*)zinc(II)

**DOI:** 10.1107/S1600536814023642

**Published:** 2014-10-31

**Authors:** Ulrich Flörke, Aziza Ahmida, Hans Egold, Gerald Henkel

**Affiliations:** a Department Chemie, Fakultät für Naturwissenschaften, Universität Paderborn, Warburgerstrasse 100, D-33098 Paderborn, Germany

**Keywords:** crystal structure, imidazoline­thio­nes, zinc(II) complex,

## Abstract

The mol­ecular structure of the title compound, [ZnCl_2_(C_11_H_20_N_2_S)_2_], shows tetra­hedral Zn coordination from two Cl ligands and two thione groups. The Zn—Cl bond lengths differ sligthly at 2.2310 (10) and 2.2396 (11) Å while the Zn—S bond lengths are equal at 2.3663 (9) and 2.3701 (10) Å. The Cl—Zn—Cl angle is 116.04 (4) and S—Zn—S is 101.98 (3)°. All other angles at the central Zn atom range from 108.108 (3) to 110.21 (4)°. The C—S—Zn angles are 100.75 (10) and 103.68 (11)°, the difference most probably resulting from packing effects, as both the C—S and both the S—Zn bonds are equal in each case. The two imidazole ring planes make a dihedral angle of 67.9 (1)°. The CH_3_ groups of one isopropyl moiety are disordered over two sets of sites with occupation factors of 0.567 (15) and 0.433 (15). It may be noteworthy that the isomolecular Cu complex shows a different crystal packing (group–subgroup relation) with the Cu atom lying on a twofold rotation axis. In the crystal, the shortest non-bonding contact is a C—H⋯Cl inter­action. This leads to the formation of centrosymmetric dimers that are stacked along the *c*-axis.

## Related literature   

For the coordination chemistry of imidazoline­thio­nes, see: Raper & Crackett (1981 ▶). For related structures, see: Williams *et al.* (1997 ▶). For the structure of the related Cu complex, see: Flörke *et al.* (2013 ▶).
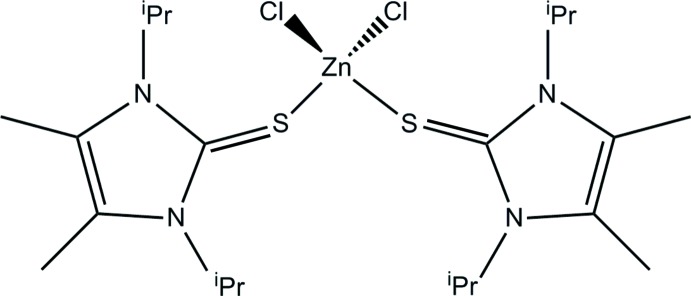


## Experimental   

### Crystal data  

[ZnCl_2_(C_11_H_20_N_2_S)_2_]
*M* *_r_* = 560.97
Monoclinic, 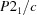

*a* = 11.997 (3) Å
*b* = 12.885 (3) Å
*c* = 18.217 (4) Å
β = 96.856 (4)°
*V* = 2796.0 (10) Å^3^
*Z* = 4
Mo *K*α radiation
μ = 1.24 mm^−1^
*T* = 120 K
0.42 × 0.28 × 0.21 mm

### Data collection  

Bruker SMART CCD area-detector diffractometer
Absorption correction: multi-scan (*SADABS*; Bruker, 2002 ▶) *T*_min_ = 0.625, *T*_max_ = 0.781
24445 measured reflections
6662 independent reflections
4453 reflections with *I* > 2σ(*I*)
*R*_int_ = 0.145

### Refinement  

*R*[*F* ^2^ > 2σ(*F* ^2^)] = 0.056
*wR*(*F* ^2^) = 0.123
*S* = 0.92
6662 reflections
293 parameters
4 restraints
H-atom parameters constrained
Δρ_max_ = 0.70 e Å^−3^
Δρ_min_ = −0.79 e Å^−3^

### 

Data collection: *SMART* (Bruker, 2002 ▶); cell refinement: *SAINT* (Bruker, 2002 ▶); data reduction: *SAINT*; program(s) used to solve structure: *SHELXTL* (Sheldrick, 2008 ▶); program(s) used to refine structure: *SHELXTL*; molecular graphics: *SHELXTL*; software used to prepare material for publication: *SHELXTL*.

## Supplementary Material

Crystal structure: contains datablock(s) I, global. DOI: 10.1107/S1600536814023642/hp2069sup1.cif

Structure factors: contains datablock(s) I. DOI: 10.1107/S1600536814023642/hp2069Isup2.hkl

**Fig. 1.**
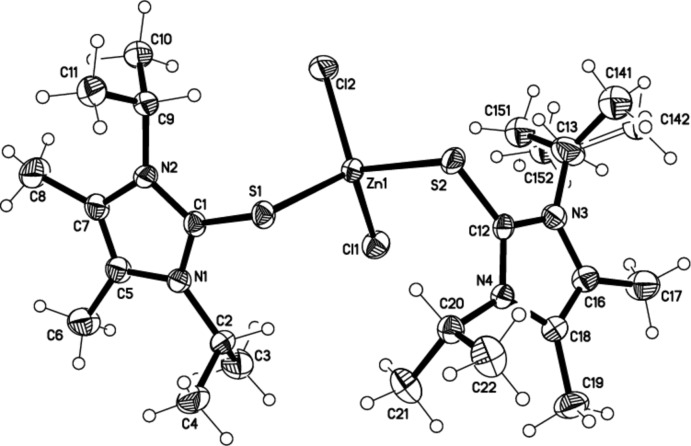
Molecular structure of the title compound with anisotropic displacement parameters drawn at the 50% probability level. Both orientations of disordered isopropyl group at C13 shown.

. DOI: 10.1107/S1600536814023642/hp2069fig1.tif
Mol­ecular structure of the title compound with anisotropic displacement parameters drawn at the 50% probability level. Both orientations of disordered isopropyl group at C13 shown.

CCDC reference: 1031227

Additional supporting information:  crystallographic information; 3D view; checkCIF report

## Figures and Tables

**Table 1:** Hydrogen-bond geometry (, )

*D*H*A*	*D*H	H*A*	*D* *A*	*D*H*A*
C17H17*A*Cl1^i^	0.98	2.78	3.745(4)	169

Symmetry code: (i) 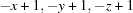.

## supplementary crystallographic information

### S1. Experimental

To a solution of 1,3-diisopropyl-4,5-dimethylimidazoline-2-thione (0.584 mg, 2.75 mmol) in acetonitrile (40 ml) ZnCl_2_ (0.171 mg, 1.25 mmol) was added and the mixture was stirred at room temperature for 24 h. Afterwards the solvent was removed under vacuum. Colourless crystals were obtained from an acetonitrile solution by diethyl ether diffusion.

### S2. Refinement

Hydrogen atoms were clearly identified in difference syntheses, refined at idealized positions riding on the carbon atoms with C–H 0.98–1.00 Å and with isotropic displacement parameters *U*_iso_(H) = 1.2*U*_eq_(C) or 1.5*U*_eq_(–CH_3_). All CH_3_ hydrogen atoms were allowed to rotate but not to tip. The disordered positions (C141/142 and C151/152) of isopropyl moiety at C13 were refined with site occupation factors 0.57 (1) and 0.43 (1), respectively and *DFIX* 1.50 0.01 restraints. Anistropic refinement of these disordered parts resulted in poor convergence, so eventually isotropic refinement was used.

### Crystal data

**Table:**

[ZnCl_2_(C_11_H_20_N_2_S)_2_]	*F*(000) = 1184
*M**_r_* = 560.97	*D*_x_ = 1.333 Mg m^−^^3^
Monoclinic, *P*2_1_/*c*	Mo *K*α radiation, λ = 0.71073 Å
*a* = 11.997 (3) Å	Cell parameters from 4648 reflections
*b* = 12.885 (3) Å	θ = 2.3–27.8°
*c* = 18.217 (4) Å	µ = 1.24 mm^−^^1^
β = 96.856 (4)°	*T* = 120 K
*V* = 2796.0 (10) Å^3^	Prism, colourless
*Z* = 4	0.42 × 0.28 × 0.21 mm

### Data collection

**Table:**

Bruker SMART CCD area-detector diffractometer	6662 independent reflections
Radiation source: sealed tube	4453 reflections with *I* > 2σ(*I*)
Graphite monochromator	*R*_int_ = 0.145
φ and ω scans	θ_max_ = 27.9°, θ_min_ = 1.7°
Absorption correction: multi-scan (*SADABS*; Bruker, 2002)	*h* = −15→15
*T*_min_ = 0.625, *T*_max_ = 0.781	*k* = −15→16
24445 measured reflections	*l* = −23→23

### Refinement

**Table:**

Refinement on *F*^2^	Primary atom site location: structure-invariant direct methods
Least-squares matrix: full	Secondary atom site location: difference Fourier map
*R*[*F*^2^ > 2σ(*F*^2^)] = 0.056	Hydrogen site location: difference Fourier map
*wR*(*F*^2^) = 0.123	H-atom parameters constrained
*S* = 0.92	*w* = 1/[σ^2^(*F*_o_^2^) + (0.045*P*)^2^] where *P* = (*F*_o_^2^ + 2*F*_c_^2^)/3
6662 reflections	(Δ/σ)_max_ = 0.001
293 parameters	Δρ_max_ = 0.70 e Å^−^^3^
4 restraints	Δρ_min_ = −0.79 e Å^−^^3^

### Special details

**Table:**

Geometry. All e.s.d.'s (except the e.s.d. in the dihedral angle between two l.s. planes) are estimated using the full covariance matrix. The cell e.s.d.'s are taken into account individually in the estimation of e.s.d.'s in distances, angles and torsion angles; correlations between e.s.d.'s in cell parameters are only used when they are defined by crystal symmetry. An approximate (isotropic) treatment of cell e.s.d.'s is used for estimating e.s.d.'s involving l.s. planes.
Refinement. Refinement of *F*^2^ against ALL reflections. The weighted *R*-factor *wR* and goodness of fit *S* are based on *F*^2^, conventional *R*-factors *R* are based on *F*, with *F* set to zero for negative *F*^2^. The threshold expression of *F*^2^ > σ(*F*^2^) is used only for calculating *R*-factors(gt) *etc*. and is not relevant to the choice of reflections for refinement. *R*-factors based on *F*^2^ are statistically about twice as large as those based on *F*, and *R*- factors based on ALL data will be even larger.

### Fractional atomic coordinates and isotropic or equivalent isotropic displacement parameters (Å^2^)

**Table:**

	*x*	*y*	*z*	*U*_iso_*/*U*_eq_	Occ. (<1)
Zn1	0.29403 (3)	0.79662 (4)	0.317723 (19)	0.02952 (12)	
Cl1	0.26708 (7)	0.64598 (7)	0.37435 (5)	0.0406 (2)	
Cl2	0.23632 (7)	0.80239 (9)	0.19670 (5)	0.0456 (2)	
S1	0.21273 (6)	0.93464 (7)	0.37853 (4)	0.0310 (2)	
S2	0.48780 (6)	0.83757 (8)	0.33891 (5)	0.0366 (2)	
N1	0.0161 (2)	0.8553 (2)	0.41595 (14)	0.0289 (6)	
N2	−0.00427 (19)	0.9400 (2)	0.31213 (14)	0.0315 (6)	
N3	0.5885 (2)	0.6531 (2)	0.38121 (14)	0.0325 (6)	
N4	0.5360 (2)	0.7405 (2)	0.47290 (14)	0.0270 (6)	
C1	0.0716 (2)	0.9057 (3)	0.36739 (17)	0.0285 (7)	
C2	0.0744 (3)	0.8058 (3)	0.48328 (17)	0.0326 (7)	
H2A	0.1558	0.8034	0.4764	0.039*	
C3	0.0380 (3)	0.6944 (4)	0.4921 (2)	0.0551 (11)	
H3A	0.0387	0.6576	0.4451	0.083*	
H3B	−0.0380	0.6932	0.5067	0.083*	
H3C	0.0899	0.6604	0.5304	0.083*	
C4	0.0658 (4)	0.8715 (4)	0.5502 (2)	0.0599 (12)	
H4A	0.0976	0.9403	0.5428	0.090*	
H4B	0.1076	0.8386	0.5936	0.090*	
H4C	−0.0132	0.8787	0.5581	0.090*	
C5	−0.0980 (3)	0.8623 (3)	0.39265 (18)	0.0355 (8)	
C6	−0.1870 (3)	0.8242 (4)	0.4368 (2)	0.0526 (11)	
H6A	−0.2606	0.8478	0.4139	0.079*	
H6B	−0.1729	0.8515	0.4873	0.079*	
H6C	−0.1857	0.7481	0.4383	0.079*	
C7	−0.1115 (2)	0.9152 (3)	0.32884 (18)	0.0359 (8)	
C8	−0.2172 (3)	0.9469 (4)	0.2828 (2)	0.0594 (13)	
H8A	−0.2814	0.9327	0.3099	0.089*	
H8B	−0.2253	0.9076	0.2365	0.089*	
H8C	−0.2143	1.0213	0.2720	0.089*	
C9	0.0265 (3)	0.9976 (3)	0.24718 (17)	0.0384 (8)	
H9A	0.1094	0.9891	0.2477	0.046*	
C10	−0.0265 (3)	0.9506 (4)	0.17534 (19)	0.0501 (10)	
H10A	−0.0198	0.8748	0.1780	0.075*	
H10B	0.0119	0.9765	0.1345	0.075*	
H10C	−0.1060	0.9699	0.1670	0.075*	
C11	0.0057 (3)	1.1130 (3)	0.2544 (2)	0.0453 (9)	
H11A	0.0528	1.1399	0.2979	0.068*	
H11B	−0.0735	1.1248	0.2600	0.068*	
H11C	0.0245	1.1488	0.2100	0.068*	
C12	0.5355 (2)	0.7400 (3)	0.39916 (17)	0.0294 (7)	
C13	0.6001 (3)	0.6225 (4)	0.3046 (2)	0.0556 (11)	
H13B	0.5685	0.6839	0.2757	0.067*	0.567 (15)
H13A	0.6363	0.5541	0.3181	0.067*	0.433 (15)
C141	0.6930 (8)	0.6679 (10)	0.2705 (5)	0.046 (3)*	0.433 (15)
H14A	0.7614	0.6660	0.3058	0.069*	0.433 (15)
H14B	0.6750	0.7400	0.2567	0.069*	0.433 (15)
H14C	0.7048	0.6281	0.2263	0.069*	0.433 (15)
C151	0.4967 (7)	0.5821 (10)	0.2609 (5)	0.042 (3)*	0.433 (15)
H15A	0.5168	0.5459	0.2170	0.062*	0.433 (15)
H15B	0.4463	0.6400	0.2456	0.062*	0.433 (15)
H15C	0.4589	0.5337	0.2912	0.062*	0.433 (15)
C142	0.7155 (5)	0.6212 (8)	0.2885 (5)	0.048 (2)*	0.567 (15)
H14D	0.7172	0.6075	0.2357	0.072*	0.567 (15)
H14E	0.7569	0.5666	0.3176	0.072*	0.567 (15)
H14F	0.7504	0.6886	0.3012	0.072*	0.567 (15)
C152	0.5241 (7)	0.5399 (7)	0.2770 (5)	0.050 (2)*	0.567 (15)
H15D	0.5194	0.5371	0.2230	0.075*	0.567 (15)
H15E	0.4494	0.5534	0.2916	0.075*	0.567 (15)
H15F	0.5522	0.4735	0.2979	0.075*	0.567 (15)
C16	0.6241 (3)	0.5981 (3)	0.44529 (18)	0.0350 (8)	
C17	0.6908 (3)	0.5006 (3)	0.4470 (2)	0.0544 (11)	
H17A	0.6978	0.4711	0.4969	0.082*	
H17B	0.7657	0.5158	0.4333	0.082*	
H17C	0.6529	0.4507	0.4118	0.082*	
C18	0.5917 (3)	0.6525 (3)	0.50254 (17)	0.0332 (7)	
C19	0.6127 (3)	0.6276 (3)	0.58272 (19)	0.0514 (10)	
H19A	0.6471	0.5587	0.5892	0.077*	
H19B	0.5414	0.6281	0.6040	0.077*	
H19C	0.6634	0.6795	0.6078	0.077*	
C20	0.4861 (3)	0.8245 (3)	0.51226 (18)	0.0331 (8)	
H20A	0.4470	0.8707	0.4732	0.040*	
C21	0.3971 (3)	0.7878 (3)	0.5579 (2)	0.0493 (10)	
H21A	0.3477	0.7382	0.5292	0.074*	
H21B	0.3529	0.8473	0.5714	0.074*	
H21C	0.4328	0.7541	0.6029	0.074*	
C22	0.5759 (3)	0.8906 (3)	0.5550 (2)	0.0528 (10)	
H22A	0.6246	0.9201	0.5209	0.079*	
H22B	0.6208	0.8476	0.5918	0.079*	
H22C	0.5404	0.9468	0.5800	0.079*	

### Atomic displacement parameters (Å^2^)

**Table:**

	*U*^11^	*U*^22^	*U*^33^	*U*^12^	*U*^13^	*U*^23^
Zn1	0.02328 (18)	0.0371 (2)	0.0277 (2)	0.00150 (16)	0.00112 (13)	0.00080 (16)
Cl1	0.0410 (5)	0.0368 (5)	0.0433 (5)	−0.0021 (4)	0.0020 (4)	0.0043 (4)
Cl2	0.0409 (5)	0.0676 (7)	0.0273 (4)	0.0026 (5)	0.0007 (3)	−0.0028 (4)
S1	0.0214 (3)	0.0376 (5)	0.0335 (4)	−0.0007 (3)	0.0004 (3)	−0.0028 (4)
S2	0.0231 (4)	0.0480 (6)	0.0378 (5)	−0.0003 (4)	0.0005 (3)	0.0150 (4)
N1	0.0253 (13)	0.0339 (16)	0.0271 (14)	−0.0022 (11)	0.0011 (10)	−0.0013 (12)
N2	0.0238 (12)	0.0441 (18)	0.0259 (14)	0.0012 (12)	0.0001 (10)	0.0036 (12)
N3	0.0281 (13)	0.0398 (17)	0.0295 (14)	−0.0035 (13)	0.0032 (11)	−0.0042 (13)
N4	0.0253 (12)	0.0271 (15)	0.0282 (14)	0.0002 (11)	0.0014 (10)	−0.0008 (11)
C1	0.0226 (14)	0.0326 (19)	0.0300 (16)	0.0007 (13)	0.0015 (12)	−0.0043 (14)
C2	0.0346 (17)	0.034 (2)	0.0284 (17)	0.0034 (15)	0.0008 (13)	0.0031 (14)
C3	0.057 (2)	0.055 (3)	0.056 (3)	0.000 (2)	0.0156 (19)	0.011 (2)
C4	0.072 (3)	0.074 (3)	0.030 (2)	0.023 (3)	−0.0079 (18)	−0.007 (2)
C5	0.0244 (15)	0.048 (2)	0.0338 (18)	−0.0043 (15)	0.0020 (13)	−0.0031 (16)
C6	0.0323 (18)	0.081 (3)	0.044 (2)	−0.013 (2)	0.0056 (16)	0.008 (2)
C7	0.0209 (14)	0.052 (2)	0.0347 (18)	−0.0015 (15)	0.0023 (12)	−0.0012 (16)
C8	0.0251 (17)	0.101 (4)	0.050 (2)	0.002 (2)	−0.0043 (15)	0.019 (2)
C9	0.0299 (16)	0.055 (2)	0.0307 (18)	0.0066 (16)	0.0053 (13)	0.0067 (16)
C10	0.050 (2)	0.068 (3)	0.0319 (19)	0.015 (2)	0.0039 (16)	0.0012 (19)
C11	0.0398 (19)	0.051 (3)	0.046 (2)	0.0043 (18)	0.0077 (16)	0.0076 (18)
C12	0.0173 (13)	0.040 (2)	0.0304 (17)	−0.0025 (13)	−0.0013 (12)	0.0023 (14)
C13	0.051 (2)	0.082 (3)	0.035 (2)	−0.010 (2)	0.0099 (17)	−0.012 (2)
C16	0.0383 (17)	0.032 (2)	0.0343 (18)	0.0013 (15)	0.0028 (14)	−0.0002 (15)
C17	0.072 (3)	0.046 (3)	0.046 (2)	0.015 (2)	0.010 (2)	0.0025 (19)
C18	0.0329 (16)	0.036 (2)	0.0299 (17)	0.0015 (15)	0.0011 (13)	0.0017 (15)
C19	0.068 (3)	0.052 (3)	0.033 (2)	0.014 (2)	0.0027 (18)	0.0075 (18)
C20	0.0338 (17)	0.031 (2)	0.0345 (18)	0.0015 (14)	0.0045 (13)	−0.0019 (14)
C21	0.042 (2)	0.052 (3)	0.057 (2)	−0.0034 (19)	0.0199 (18)	−0.008 (2)
C22	0.046 (2)	0.047 (3)	0.066 (3)	−0.0073 (19)	0.0091 (19)	−0.017 (2)

### Geometric parameters (Å, º)

**Table:**

Zn1—Cl2	2.2319 (10)	C10—H10C	0.9800
Zn1—Cl1	2.2396 (10)	C11—H11A	0.9800
Zn1—S1	2.3663 (9)	C11—H11B	0.9800
Zn1—S2	2.3701 (10)	C11—H11C	0.9800
S1—C1	1.722 (3)	C13—C152	1.451 (7)
S2—C12	1.722 (3)	C13—C141	1.461 (7)
N1—C1	1.337 (4)	C13—C142	1.449 (6)
N1—C5	1.388 (4)	C13—C151	1.486 (7)
N1—C2	1.482 (4)	C13—H13B	1.0000
N2—C1	1.349 (4)	C13—H13A	0.9999
N2—C7	1.394 (4)	C141—H14A	0.9800
N2—C9	1.480 (4)	C141—H14B	0.9800
N3—C12	1.347 (4)	C141—H14C	0.9800
N3—C16	1.388 (4)	C151—H15A	0.9800
N3—C13	1.473 (4)	C151—H15B	0.9800
N4—C12	1.343 (4)	C151—H15C	0.9800
N4—C18	1.392 (4)	C142—H14D	0.9800
N4—C20	1.466 (4)	C142—H14E	0.9800
C2—C4	1.498 (5)	C142—H14F	0.9800
C2—C3	1.514 (6)	C152—H15D	0.9800
C2—H2A	1.0000	C152—H15E	0.9800
C3—H3A	0.9800	C152—H15F	0.9800
C3—H3B	0.9800	C16—C18	1.351 (5)
C3—H3C	0.9800	C16—C17	1.488 (5)
C4—H4A	0.9800	C17—H17A	0.9800
C4—H4B	0.9800	C17—H17B	0.9800
C4—H4C	0.9800	C17—H17C	0.9800
C5—C7	1.341 (5)	C18—C19	1.488 (5)
C5—C6	1.494 (5)	C19—H19A	0.9800
C6—H6A	0.9800	C19—H19B	0.9800
C6—H6B	0.9800	C19—H19C	0.9800
C6—H6C	0.9800	C20—C21	1.506 (5)
C7—C8	1.491 (4)	C20—C22	1.513 (5)
C8—H8A	0.9800	C20—H20A	1.0000
C8—H8B	0.9800	C21—H21A	0.9800
C8—H8C	0.9800	C21—H21B	0.9800
C9—C10	1.512 (5)	C21—H21C	0.9800
C9—C11	1.516 (6)	C22—H22A	0.9800
C9—H9A	1.0000	C22—H22B	0.9800
C10—H10A	0.9800	C22—H22C	0.9800
C10—H10B	0.9800		
			
Cl2—Zn1—Cl1	116.04 (4)	N3—C12—S2	125.7 (2)
Cl2—Zn1—S1	109.93 (4)	C152—C13—C141	128.8 (5)
Cl1—Zn1—S1	110.21 (4)	C152—C13—C142	119.8 (6)
Cl2—Zn1—S2	109.64 (3)	C141—C13—C142	28.7 (4)
Cl1—Zn1—S2	108.10 (3)	C152—C13—N3	113.4 (4)
S1—Zn1—S2	101.98 (3)	C141—C13—N3	117.3 (5)
C1—S1—Zn1	103.68 (11)	C142—C13—N3	113.3 (4)
C12—S2—Zn1	100.75 (10)	C152—C13—C151	26.9 (4)
C1—N1—C5	108.5 (3)	C141—C13—C151	122.9 (7)
C1—N1—C2	122.3 (2)	C142—C13—C151	130.1 (5)
C5—N1—C2	129.2 (3)	N3—C13—C151	115.8 (4)
C1—N2—C7	108.7 (3)	C152—C13—H13B	102.4
C1—N2—C9	123.4 (2)	C141—C13—H13B	73.8
C7—N2—C9	127.9 (3)	C142—C13—H13B	102.4
C12—N3—C16	109.0 (3)	N3—C13—H13B	102.4
C12—N3—C13	123.6 (3)	C151—C13—H13B	75.8
C16—N3—C13	127.3 (3)	C152—C13—H13A	71.1
C12—N4—C18	109.2 (3)	C141—C13—H13A	97.3
C12—N4—C20	122.7 (3)	C142—C13—H13A	69.1
C18—N4—C20	128.1 (3)	N3—C13—H13A	95.2
N1—C1—N2	108.0 (2)	C151—C13—H13A	97.7
N1—C1—S1	126.0 (2)	H13B—C13—H13A	162.4
N2—C1—S1	125.7 (2)	C13—C141—H14A	109.5
N1—C2—C4	111.0 (3)	C13—C141—H14B	109.5
N1—C2—C3	112.5 (3)	C13—C141—H14C	109.5
C4—C2—C3	113.6 (3)	C13—C151—H15A	109.5
N1—C2—H2A	106.4	C13—C151—H15B	109.5
C4—C2—H2A	106.4	C13—C151—H15C	109.5
C3—C2—H2A	106.4	C13—C142—H13A	40.5
C2—C3—H3A	109.5	C13—C142—H14D	109.5
C2—C3—H3B	109.5	C13—C142—H14E	109.5
H3A—C3—H3B	109.5	H14D—C142—H14E	109.5
C2—C3—H3C	109.5	C13—C142—H14F	109.5
H3A—C3—H3C	109.5	H14D—C142—H14F	109.5
H3B—C3—H3C	109.5	H14E—C142—H14F	109.5
C2—C4—H4A	109.5	C13—C152—H13A	40.0
C2—C4—H4B	109.5	C13—C152—H15D	109.5
H4A—C4—H4B	109.5	C13—C152—H15E	109.5
C2—C4—H4C	109.5	H15D—C152—H15E	109.5
H4A—C4—H4C	109.5	C13—C152—H15F	109.5
H4B—C4—H4C	109.5	H15D—C152—H15F	109.5
C7—C5—N1	108.1 (3)	H15E—C152—H15F	109.5
C7—C5—C6	127.9 (3)	C18—C16—N3	107.3 (3)
N1—C5—C6	123.8 (3)	C18—C16—C17	128.7 (3)
C5—C6—H6A	109.5	N3—C16—C17	123.9 (3)
C5—C6—H6B	109.5	C16—C17—H17A	109.5
H6A—C6—H6B	109.5	C16—C17—H17B	109.5
C5—C6—H6C	109.5	H17A—C17—H17B	109.5
H6A—C6—H6C	109.5	C16—C17—H17C	109.5
H6B—C6—H6C	109.5	H17A—C17—H17C	109.5
C5—C7—N2	106.7 (3)	H17B—C17—H17C	109.5
C5—C7—C8	129.3 (3)	C16—C18—N4	107.0 (3)
N2—C7—C8	124.0 (3)	C16—C18—C19	128.0 (3)
C7—C8—H8A	109.5	N4—C18—C19	125.0 (3)
C7—C8—H8B	109.5	C18—C19—H19A	109.5
H8A—C8—H8B	109.5	C18—C19—H19B	109.5
C7—C8—H8C	109.5	H19A—C19—H19B	109.5
H8A—C8—H8C	109.5	C18—C19—H19C	109.5
H8B—C8—H8C	109.5	H19A—C19—H19C	109.5
N2—C9—C10	111.8 (3)	H19B—C19—H19C	109.5
N2—C9—C11	111.3 (3)	N4—C20—C21	113.3 (3)
C10—C9—C11	114.3 (3)	N4—C20—C22	111.1 (3)
N2—C9—H9A	106.3	C21—C20—C22	113.6 (3)
C10—C9—H9A	106.3	N4—C20—H20A	106.0
C11—C9—H9A	106.3	C21—C20—H20A	106.0
C9—C10—H10A	109.5	C22—C20—H20A	106.0
C9—C10—H10B	109.5	C20—C21—H21A	109.5
H10A—C10—H10B	109.5	C20—C21—H21B	109.5
C9—C10—H10C	109.5	H21A—C21—H21B	109.5
H10A—C10—H10C	109.5	C20—C21—H21C	109.5
H10B—C10—H10C	109.5	H21A—C21—H21C	109.5
C9—C11—H11A	109.5	H21B—C21—H21C	109.5
C9—C11—H11B	109.5	C20—C22—H22A	109.5
H11A—C11—H11B	109.5	C20—C22—H22B	109.5
C9—C11—H11C	109.5	H22A—C22—H22B	109.5
H11A—C11—H11C	109.5	C20—C22—H22C	109.5
H11B—C11—H11C	109.5	H22A—C22—H22C	109.5
N4—C12—N3	107.5 (3)	H22B—C22—H22C	109.5
N4—C12—S2	126.6 (3)		
			
Cl2—Zn1—S1—C1	65.79 (12)	C7—N2—C9—C11	75.6 (4)
Cl1—Zn1—S1—C1	−63.33 (12)	C18—N4—C12—N3	0.7 (3)
S2—Zn1—S1—C1	−177.95 (11)	C20—N4—C12—N3	179.5 (3)
Cl2—Zn1—S2—C12	−133.06 (12)	C18—N4—C12—S2	−173.9 (2)
Cl1—Zn1—S2—C12	−5.69 (12)	C20—N4—C12—S2	4.9 (4)
S1—Zn1—S2—C12	110.47 (12)	C16—N3—C12—N4	−0.6 (3)
C5—N1—C1—N2	−2.9 (4)	C13—N3—C12—N4	176.9 (3)
C2—N1—C1—N2	179.0 (3)	C16—N3—C12—S2	174.1 (2)
C5—N1—C1—S1	170.2 (3)	C13—N3—C12—S2	−8.4 (4)
C2—N1—C1—S1	−7.9 (5)	Zn1—S2—C12—N4	−85.6 (3)
C7—N2—C1—N1	3.4 (4)	Zn1—S2—C12—N3	100.7 (3)
C9—N2—C1—N1	−178.7 (3)	C12—N3—C13—C152	−103.5 (6)
C7—N2—C1—S1	−169.8 (3)	C16—N3—C13—C152	73.6 (7)
C9—N2—C1—S1	8.1 (5)	C12—N3—C13—C141	84.0 (8)
Zn1—S1—C1—N1	95.2 (3)	C16—N3—C13—C141	−99.0 (8)
Zn1—S1—C1—N2	−92.8 (3)	C12—N3—C13—C142	115.5 (6)
C1—N1—C2—C4	101.7 (4)	C16—N3—C13—C142	−67.4 (7)
C5—N1—C2—C4	−76.0 (5)	C12—N3—C13—C151	−74.0 (8)
C1—N1—C2—C3	−129.7 (3)	C16—N3—C13—C151	103.1 (7)
C5—N1—C2—C3	52.6 (5)	C12—N3—C16—C18	0.2 (4)
C1—N1—C5—C7	1.3 (4)	C13—N3—C16—C18	−177.2 (3)
C2—N1—C5—C7	179.3 (3)	C12—N3—C16—C17	−176.8 (3)
C1—N1—C5—C6	−173.8 (4)	C13—N3—C16—C17	5.9 (5)
C2—N1—C5—C6	4.2 (6)	N3—C16—C18—N4	0.2 (4)
N1—C5—C7—N2	0.8 (4)	C17—C16—C18—N4	177.0 (3)
C6—C5—C7—N2	175.6 (4)	N3—C16—C18—C19	−178.1 (3)
N1—C5—C7—C8	−177.6 (4)	C17—C16—C18—C19	−1.4 (6)
C6—C5—C7—C8	−2.8 (7)	C12—N4—C18—C16	−0.6 (4)
C1—N2—C7—C5	−2.6 (4)	C20—N4—C18—C16	−179.2 (3)
C9—N2—C7—C5	179.6 (3)	C12—N4—C18—C19	177.8 (3)
C1—N2—C7—C8	175.9 (4)	C20—N4—C18—C19	−0.8 (5)
C9—N2—C7—C8	−1.9 (6)	C12—N4—C20—C21	122.8 (3)
C1—N2—C9—C10	128.9 (3)	C18—N4—C20—C21	−58.7 (4)
C7—N2—C9—C10	−53.6 (5)	C12—N4—C20—C22	−107.9 (3)
C1—N2—C9—C11	−101.9 (4)	C18—N4—C20—C22	70.6 (4)

### Hydrogen-bond geometry (Å, º)

**Table:**

*D*—H···*A*	*D*—H	H···*A*	*D*···*A*	*D*—H···*A*
C17—H17*A*···Cl1^i^	0.98	2.78	3.745 (4)	169

Symmetry code: (i) −*x*+1, −*y*+1, −*z*+1.
