# Supplementary figures and images for: Crystal structure of di­chlorido­bis­(1,3-diisopropyl-4,5-dimethyl-2H-imidazole-2-thione-κS)zinc(II)
Source: Acta Crystallogr Sect E Struct Rep Online. 2014 Oct 31;70(Pt 11):m384. doi: 10.1107/S1600536814023642 (PMC4257301; doi:10.1107/S1600536814023642)

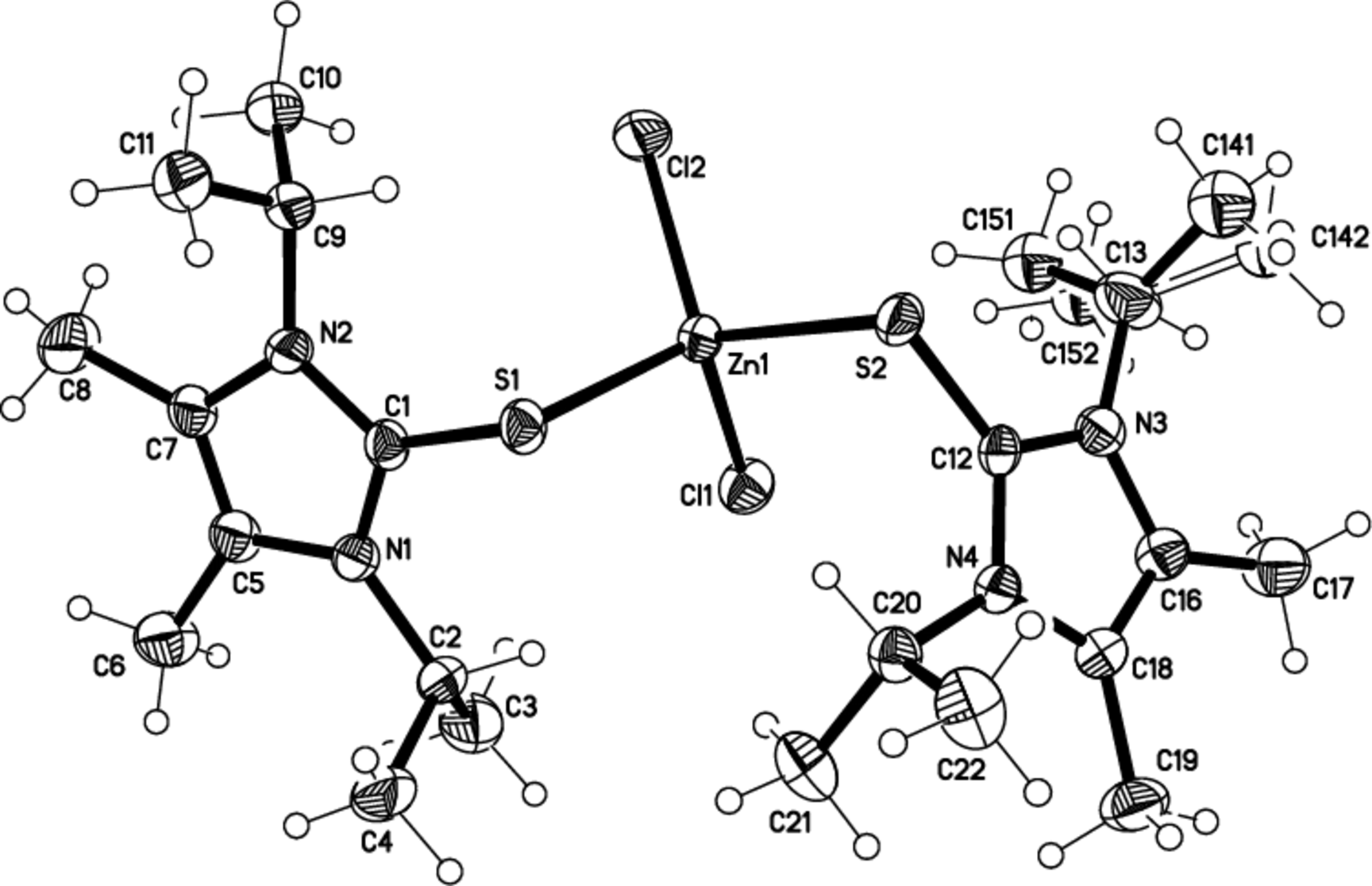

Supplement: Supplementary file 3 [file e-70-0m384-fig1.tif]
